# Quantifying the effects of antibiotic treatment on the extracellular polymer network of antimicrobial resistant and sensitive biofilms using multiple particle tracking

**DOI:** 10.1038/s41522-020-00172-6

**Published:** 2021-02-05

**Authors:** Lydia C. Powell, Muthanna Abdulkarim, Joana Stokniene, Qiu E. Yang, Timothy R. Walsh, Katja E. Hill, Mark Gumbleton, David W. Thomas

**Affiliations:** 1 Advanced Therapies Group, Cardiff University School of Dentistry, Cardiff, UK; 2 Centre of Nanohealth, Swansea University Medical School, Swansea University, Swansea, UK; 3 School of Pharmacy and Pharmaceutical Sciences, Cardiff University, Cardiff, UK; 4 Medical Microbiology and Infectious Disease, School of Medicine, Cardiff University, Cardiff, UK

**Keywords:** Biofilms, Biological techniques

## Abstract

Novel therapeutics designed to target the polymeric matrix of biofilms requires innovative techniques to accurately assess their efficacy. Here, multiple particle tracking (MPT) was developed to characterize the physical and mechanical properties of antimicrobial resistant (AMR) bacterial biofilms and to quantify the effects of antibiotic treatment. Studies employed nanoparticles (NPs) of varying charge and size (40–500 nm) in *Pseudomonas aeruginosa* PAO1 and methicillin-resistant *Staphylococcus aureus* (MRSA) biofilms and also in polymyxin B (PMB) treated *Escherichia coli* biofilms of PMB-sensitive (PMB^Sens^) IR57 and PMB-resistant (PMB^R^) PN47 strains. NP size-dependent and strain-related differences in the diffusion coefficient values of biofilms were evident between PAO1 and MRSA. Dose-dependent treatment effects induced by PMB in PMB^Sens^ *E*. *coli* biofilms included increases in diffusion and creep compliance (*P* < 0.05), not evident in PMB treatment of PMB^R^ *E. coli* biofilms. Our results highlight the ability of MPT to quantify the diffusion and mechanical effects of antibiotic therapies within the AMR biofilm matrix, offering a valuable tool for the pre-clinical screening of anti-biofilm therapies.

## Introduction

The important role of bacterial biofilms in chronic human diseases, such as cystic fibrosis, otitis media, chronic skin wounds and implant- and catheter-associated infections, has been increasingly recognized^1^. Within these biofilms, the bacteria are embedded in a complex, charged, self-produced extracellular polymeric matrix (EPS). The EPS matrix is an entangled polymer network^2^ predominately composed of polysaccharides, extracellular DNA (eDNA), proteins and lipids, which facilitates biofilm formation and maturation^3^. The matrix confers considerable fitness advantages over planktonic bacteria in hydration, protection from environmental tensile or shear forces, increased cell–cell communication and enhanced horizontal gene transfer^4^. The EPS matrix also offers protection from antimicrobials, as bacteria within biofilm structures can resist conventional antibiotic therapies up to 10^3^-fold^5–7^. This inherent ability of biofilms to resist antibiotics occurs through reduced metabolic activity, development of persister cells^8^ and reduced antibiotic/small molecular diffusion through the EPS polymeric network via charge-interactions^9–11^.

The global rise of antibiotic resistance is an increasing problem, threatening the ability of healthcare providers to treat common bacterial infections, such as hospital-acquired methicillin-resistant *Staphylococcus aureus* (MRSA)^12^. Multidrug resistance in bacteria is also of increasing concern due to their ability to acquire multiple resistance mechanisms through horizontal gene transfer. More worrying still is the emergence of resistance to the so called ‘antibiotics of last resort;’ i.e. the polypeptide polymyxin antibiotics^13,14^, particularly those carried on mobile genetic elements such as plasmids. The colistin-resistant *mcr-1* gene is most often isolated in *E. coli*^15,16^, although recently it has also been found to have spread to *Klebsiella* sp. carried on a broad host range, self-transferable IncP plasmid^17^ suggesting the high likelihood of imminent further spread to other Gram-negative species.

Modification of biofilm assembly and targeted disruption of the cross-linked network of “entangled polymers” within the biofilm EPS matrix would be advantageous in both clinical and industrial applications, especially with the global rise of antibiotic resistance. Most strategies fall into one of two categories; biofilm disruption or biofilm prevention. To date, anti-biofilm strategies have amongst others, included surface/substrate modification (to modify initial bacterial adhesion)^18^, disruption of the biofilm matrix using conventional antibiotics^19^, antimicrobial peptides^20^, dispersal agents^21^, detergents^22^, chelators (e.g., EDTA)^23^, EPS synthesis inhibitors^24^, and dysregulation of quorum sensing within biofilms^25,26^. In attempting to design and deliver new antimicrobial and anti-biofilm therapies, the ability to accurately measure the effects of such potential therapies upon the biofilm and biofilm matrix, and accurately quantify the complexity and variability of these biofilms is currently challenging. Conventional biofilm characterization techniques include scanning electron microscopy (SEM) and confocal laser scanning microscopy (CLSM), often combined with image analysis. These techniques, however, fail to accurately characterize the EPS structure of treated biofilms, due to extensive sample preparation and/or sample dehydration, or the lack of universal dyes for EPS staining^27^. Moreover, the EPS biofilm matrix varies depending on species, strain and growth environment, for example, *P. aeruginosa* produces different types of polysaccharides with varying charge namely, cationic Pel and neutral Psl, as well as anionic alginate^28–30^ which further complicates characterization.

To understand the key ‘fitness’ advantages that biofilms possess against antimicrobial treatment, workers have sought to characterize the diffusion and material properties of the whole biofilm matrix using mesoscale and nanoscale technologies, without the need to visualize the actual EPS matrix itself. Such technologies include shear and extensional rheology^31,32^, fluorescence correlation spectroscopy (FCS)^33,34^ and fluorescence recovery after photobleaching (FRAP)^35^. Both FCS and FRAP employ fluorescently-labelled particles which can be traced within the biofilm structures. In FRAP, an area of biofilm is photo-bleached and fluorescence recovery (into the area) is modelled to determine diffusion parameters, whereas FCS is based on diffusion measurements from single-molecule fluorescence intensity fluctuations within a discrete region of the biofilm^34,36^.

Multiple particle tracking (MPT) is a recently described technique, allowing simultaneous tracking of nano-sized particles using fluorescent microscopy, from which the diffusion-based parameters of embedded particles within the EPS of bacterial biofilms can be determined^37^. MPT also facilitates measurement of the micro-rheological properties of biofilms^38^. MPT, when used in conjunction with nanoparticles (NPs) of discrete size and charge, represents a non-invasive technique which can be readily employed in situ within biofilms. MPT has subsequently been employed to characterize the diffusion properties of NPs within biofilms of a range of bacterial species including *P. aeruginosa, E. coli*, *P. fluorescens* and *S. aureus* and also to determine time-dependent changes in the biofilm matrix following adhesion^36,37,39,40^.

In this study, we sought to develop the MPT biofilm model to detect variations in the biofilm structure of Gram-negative *P. aeruginosa* and Gram-positive *S. aureus* biofilms and examine its sensitivity and correlation to CLSM imaging. Using polymyxin B-sensitive (PMB^Sens^) and resistant (PMB^R^) *E. coli* strains, we examined the sensitivity of the MPT biofilm model to detect variations in the biofilm structure after dose-dependent polymyxin B antibiotic therapy in comparison to traditional confocal microscopy. By using commercially available NPs, this study highlights the usefulness and sensitivity of the MPT technique in the development of novel anti-biofilm therapeutics for AMR infections.

## Results

### CLSM imaging and MPT measurements identify distinct variations in *S. aureus* and *P. aeruginosa* biofilms

CLSM imaging of *S. aureus* 1004A (MRSA) and *P. aeruginosa* PAO1 revealed variation in the structural properties of the biofilms produced by the two strains. While MRSA formed a thin, but bacterially-dense biofilm structure, PAO1 biofilms possessed greater height, but appeared less bacterially-dense (Supplementary Fig. 1). These CLSM images correlated well with diffusion of the negatively charged NPs (40–500 nm) through the biofilm, with NP diffusion being lower in the MRSA biofilms when compared to those of PAO1 (40 and 200 nm NPs; *P* < 0.01). In contrast, diffusion of the positively charged NPs (200 nm) in both biofilms were similar (*P* > 0.05; Table 1).

**Table 1:** NP diffusion within *P. aeruginosa* PAO1 and *S. aureus* 1004A (MRSA) biofilm structures.

*Fluo*Sphere®	*Fluo*Sphere® size (nm)* Mean (PDI)	*Fluo*Sphere® zeta potential (mV) Mean (±SEM)	Diffusion coefficient in water (D°) (cm^2^/s × 10^−9^)	Diffusion coefficient <Deff>_PAO1_ (cm^2^/s × 10^−9^) Mean (±SEM)	% ratio <Deff>_PAO1_ /D°	Diffusion coefficient <Deff>_MRSA_ (cm^2^/s × 10^−9^) Mean (±SEM)	% ratio <Deff>_MRSA_ /D°
−ve carboxylate	54.73 (0.08)	−37.86 (±0.85)	82.11	3.1493 (±0.6071)	3.8352	1.1584 (±0.1945)	1.4107
−ve carboxylate	108.9 (0.026)	−40.96 (±2.05)	41.27	1.6647 (±0.3695)	4.0338	0.3549 (±0.0624)	0.8600
−ve carboxylate	239.4 (0.031)	−40.83 (±1.30)	18.77	0.0358 (±0.0067)	0.1907	0.0031 (±0.0006)	0.0165
−ve carboxylate	517.2 (0.059)	−40.83 (±2.07)	8.69	0.0165 (±0.0038)	0.1899	0.0038 (±0.0007)	0.0437
+ve amine	204.2 (0.029)	+8.90 (±0.53)	22.01	0.0061 (±0.0015)	0.0277	0.0074 (±0.0017)	0.0336

Diffusion coefficients of 40, 100, 200 and 500 nm negatively charged carboxylate-modified *Fluo*Spheres® and 200 nm positively charged amine-modified *Fluo*Spheres® in water calculated by Stoke–Einstein equation versus their effective diffusion coefficients through *P. aeruginosa* PAO1 and *S. aureus* 1004A (MRSA) biofilms measured by the MPT technique. *Independent assessment of *Fluo*Sphere size.

<Deff>_PAO1_ and <Deff>_MRSA_ indicate <Deff> in *P. aeruginosa* PAO1 and *S*. *aureus* MRSA biofilms, respectively.

*PDI* polydispersity index.

± represents standard error of the mean (SEM; *n* = 3).

MPT revealed that as the size of the negatively charged particles increased, the diffusion coefficient of the particles within PAO1 biofilms decreased significantly (40–200 nm; *P* < 0.05). This significant trend was also evident in the MRSA biofilms for particle sizes of 40–200 nm. Also, while diffusion of the positively charged 200 nm NPs in PAO1 biofilms was significantly reduced when compared with the diffusion of the negatively charged NPs of the same size (*P* < 0.05), in MRSA biofilms, the diffusion of the positively charged NPs was not significantly different when compared to the negatively charged particles (*P* > 0.05; Table 1).

The ratio of the biofilm diffusion coefficient to the diffusion coefficient in water (<Deff>/D°) allows measurement of NP diffusion through the biofilm structure in relation to the intrinsic free Brownian motion of the NPs in water, thereby taking into account the impact of NP size on its unrestricted diffusion in liquid. The percentage ratio of <Deff>/D° revealed that the diffusion of 200 and 500 nm sized NPs were significantly lower than the diffusion of 40 and 100 nm NPs in both MRSA and PAO1 biofilms (*P* < 0.05). However, negatively charged NP diffusion was still greater (by at least 2.5 times) in PAO1 biofilms when compared to their diffusion in MRSA biofilms (Table 1).

**Fig. 1:**
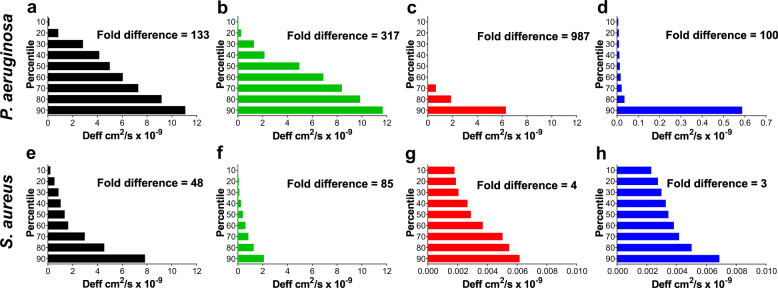
Heterogeneity of NP movement through *P. aeruginosa* PAO1 and *S. aureus* 1004A (MRSA) biofilm structures. Heterogeneity of 40, 100, 200 and 500 nm negatively charged carboxylate-modified *Fluo*Sphere® NP movement through *P. aeruginosa* and *S. aureus* biofilms. For each particle type, the effective diffusion coefficient ratio of <Deff> was calculated for 360 individual particles (*n* = 3 for biofilm experiments, each comprised of 120 particles) over a time interval of 20 s and then data was ranked into percentiles from the 90th through to 10th percentile. **a**, **e** Negatively charged 40 nm carboxylate-modified *Fluo*Sphere® in *P. aeruginosa* and *S. aureus* biofilms, respectively; **b**, **f** negatively charged 100 nm carboxylate-modified *Fluo*Sphere® in *P. aeruginosa* and *S. aureus* biofilms, respectively; **c**, **g** negatively charged 200 nm carboxylate-modified *Fluo*Sphere® in *P. aeruginosa* and *S. aureus* biofilms, respectively; **d**, **h** negatively charged 500 nm carboxylate-modified *Fluo*Sphere® in *P. aeruginosa* and *S. aureus* biofilms, respectively. Fold difference in the figure indicates the fold difference between the 90th and 10th percentiles.

PAO1 biofilms displayed greater heterogeneity in negatively charged NP movement with resultant 90th/10th percentile ratios of 100–987, when compared to MRSA biofilms which displayed much lower ratios of 3–85 (Fig. 1). The heterogeneity data again confirmed the observed variations in the structural properties between MRSA and PAO1 biofilms seen in the CLSM images. The data also revealed that there was increasing heterogeneity in NP diffusion with increasing particle size until the NP size reached 100 nm for *S. aureus* and 200 nm for *P. aeruginosa* biofilm systems, after which heterogeneity in NP diffusion then decreased.

**Fig. 2:**
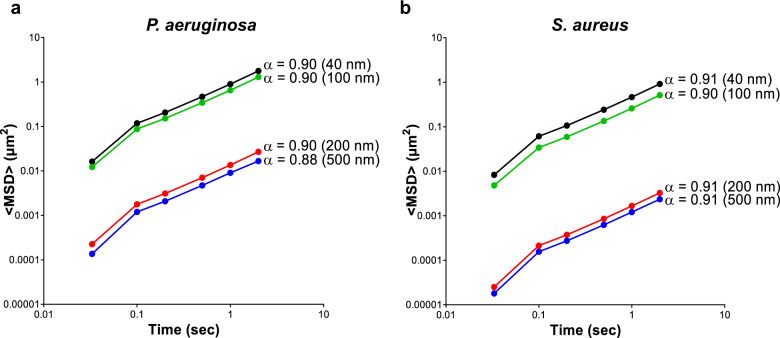
Exponential anomalous values <*α*> of *P. aeruginosa* PAO1 and *S. aureus* 1004A (MRSA) biofilms. Exponential anomalous values <*α*> of **a** *P. aeruginosa* and **b** *S. aureus* biofilms using 40, 100, 200 and 500 nm negatively charged carboxylate-modified *Fluo*Spheres®. *α* is measured based on the relation between the ensemble mean square displacement <MSD> versus time scale of the traced *Fluo*Sphere® particles and reflects the micro-rheological degree of resistance of the biofilm towards traced particles where; *α* > 0.5 indicates viscous resistance; *α* < 0.5 indicates elastic resistance. <MSD> represents the geometric mean of MSDs of 360 particles (*n* = 3, each 120 particles).

The exponential anomalous values of both PAO1 and MRSA biofilms calculated using 40–500 nm NPs appeared to demonstrate a viscous response of these biofilms (*α* > 0.5; Fig. 2).

### CLSM and MPT measurements describe dose-dependent disruption of *E. coli* IR57 biofilms treated with polymyxin B

MIC assays of PMB^Sens^ *E. coli* IR57 and PMB^R^ *E. coli* PN47 were performed to confirm strain sensitivity to the antibiotic, giving MIC values against polymyxin B of 0.06 and 2 µg/ml, respectively.

**Fig. 3:**
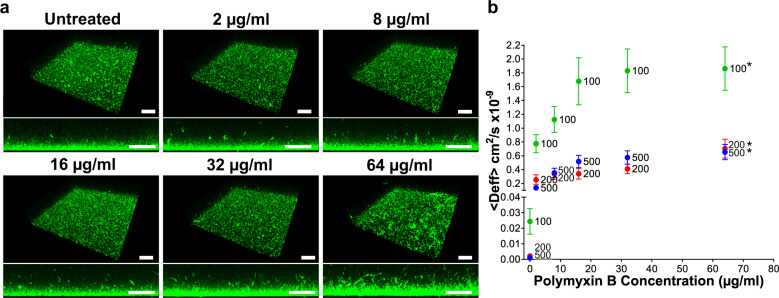
Nanoparticle diffusion within and disruption of *E. coli* IR57 (PMB^Sens^) biofilms treated with Polymyxin B. Polymyxin B (PMB)-treated (2, 8, 16, 32, 64 µg/ml) and untreated PMB^Sens^ *E. coli* IR57 biofilms showing: **a** CLSM 3D and side-on imaging of biofilms grown for 48 h followed by polymyxin B treatment for a further 24 h at 37 °C, visualized using Syto9® staining (scale bar, 40 µm; *n* = 3). **b** Diffusion coefficient <Deff> of 100, 200 and 500 nm negatively charged carboxylate-modified *Fluo*Sphere® particles in non-treated versus polymyxin B treated biofilms (*n* = 3, ± SEM). *indicates *Fluo*Sphere® particle sizes of 100, 200 and 500 nm.

The CLSM assay revealed cellular aggregation and disruption of the PMB^Sens^ *E. coli* IR57 biofilm matrix in a dose-dependent manner following polymyxin B treatment (Fig. 3a). This finding was confirmed by MPT through dose-dependent increases in the effective diffusion coefficients (<Deff>) within the treated biofilms for all three NP sizes tested (100, 200 and 500 nm; Fig. 3), with the 100 nm NPs revealing the greatest dose-dependent sensitivity in diffusion coefficients to antibiotic treatment. The dose-dependent changes in NP diffusion coefficients, starting from 2 µg/ml polymyxin B treatment, were all significantly different from the control (*P* < 0.05).

**Fig. 4:**
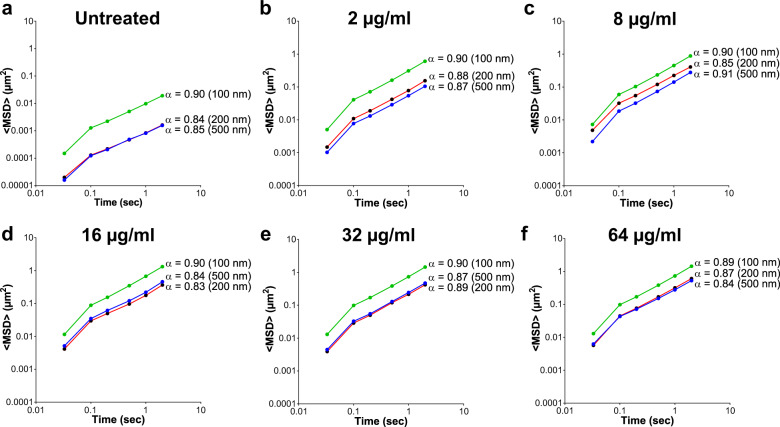
Exponential anomalous values <*α*> of *E. coli* IR57 (PMB^Sens^) biofilms treated with Polymyxin B. Exponential anomalous values <*α*> of *E. coli* IR57 biofilms in response to polymyxin B treatment using 100, 200 and 500 nm negatively charged carboxylate-modified *Fluo*Spheres® **a** untreated control; **b** 2; **c**, 8; **d**, 16; **e**, 32; and **f** 64 µg/ml polymyxin B treatment. α is measured based on the relation between the ensemble mean square displacement (MSD) versus time scale of the traced *Fluo*Sphere® particles and reflects the micro-rheological degree of resistance of the biofilm towards traced particles where; *α* > 0.5 indicates viscous resistance; *α* < 0.5 indicates elastic resistance. <MSD> represents the geometric mean of MSDs of 360 particles (*n* = 3, each 120 particles).

**Fig. 5:**
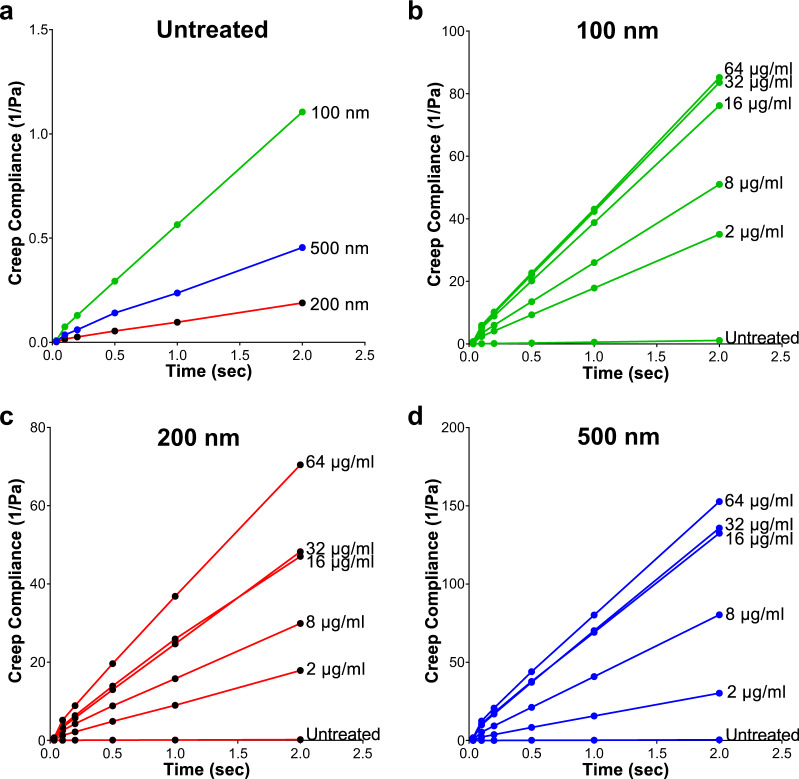
Creep compliance of *E. coli* IR57 (PMB^Sens^) biofilms treated with Polymyxin B. Creep compliance (*J*(*t*)) of *E. coli* IR57 (PMB^Sens^) biofilms (measured using the ensemble mean square displacement <MSD> versus lag time of 100, 200 and 500 nm negatively charged carboxylate-modified *Fluo*Sphere® particles) representing biofilm deformation in response to polymyxin B treatment (2, 8, 16, 32 and 64 µg/ml) versus untreated biofilm. **a** Untreated *E. coli* biofilms for 100, 200, and 500 nm *Fluo*Sphere® particles, **b** 100 nm *Fluo*Sphere® particles, **c** 200 nm *Fluo*Sphere® particles, and **d** 500 nm *Fluo*Sphere® particles, in response to polymyxin B treatment.

Polymyxin B treatment (2–8 µg/ml) revealed a greater increase in the mean square displacement <MSD> versus time measurements for the 200 and 500 nm particles when compared to the 100 nm particles, indicative of increasing pore size within the biofilm structure following polymyxin B treatment (Fig. 4). The exponential anomalous values of the *E. coli* biofilms demonstrated that polymyxin B treatment had little effect on the viscoelastic response of the biofilms (Fig. 4). However, the dose-dependent disruption of the *E. coli* IR57 biofilms was reflected in increasing biofilm creep compliance (*J*(*t*)) with increasing polymyxin B dose, observed with all 3 NP sizes tested (Fig. 5).

### MPT measurements describe distinct variations in the response of PMB^Sens^*E. coli* IR57 and PMB^R^*E. coli* PN47 biofilms to polymyxin B treatment

**Fig. 6:**
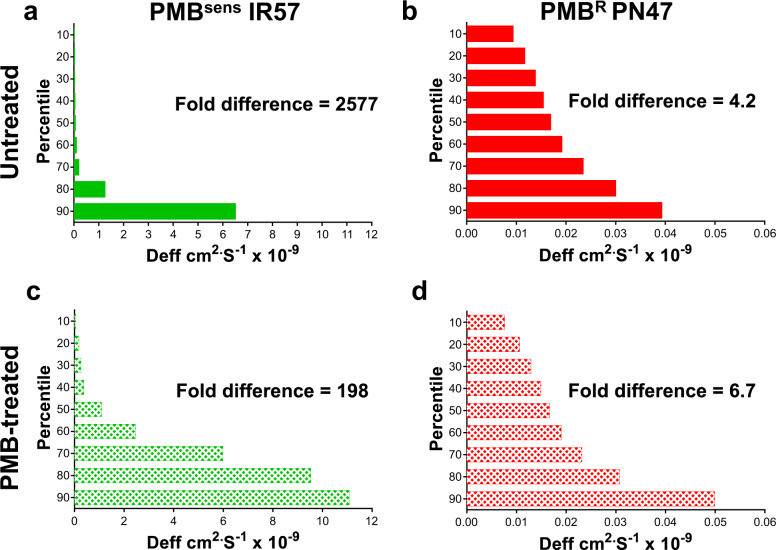
Heterogeneity of NP movement through PMB^Sens^*E. coli* IR57 and PMB^R^*E. coli* PN47 biofilm structures ± Polymyxin B treatment. Heterogeneity of 200 nm negatively charged carboxylate-modified *Fluo*Spheres® movement within *E. coli* biofilms. Untreated controls **a** PMB^Sens^ *E. coli* IR57, **b** PMB^R^ *E. coli* PN47. Polymyxin B (PMB; 8 µg/ml) treated biofilms, **c** PMB^sens^ *E. coli* IR57, **d** PMB^R^ *E. coli* PN47. For each particle type, the effective diffusion coefficient <Deff> was calculated for 360 individual particles (*n* = 3 for biofilm experiments, each comprised of 120 particles) over a time interval of 20 s and the data ranked into percentiles (90th to 10th). Fold difference in the figure indicates the fold difference between the 90th and 10th percentiles.

**Fig. 7:**
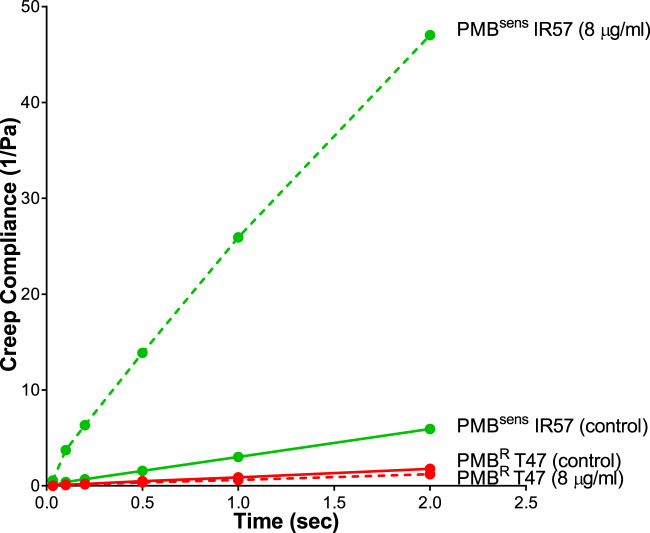
Creep compliance of PMB^Sens^*E. coli* IR57 and PMB^R^*E. coli* PN47 biofilm structures ± Polymyxin B treatment. Creep compliance (*J*(*t*)) of polymyxin B (PMB, 8 µg/ml) treated (dashed lines) and untreated control (solid lines) *E. coli* IR57 and PN47 biofilms (measured using ensemble mean square displacement <MSD> versus time of 200 nm negatively charged carboxylate-modified *Fluo*Sphere® particles).

To assess the impact of resistance to polymyxin B on treatment of *E. coli* biofilms, two strains of *E. coli* (PMB^R^ PN47 and PMB^Sens^ IR57) were selected based on their susceptibility to polymyxin B. The MPT assay revealed that the diffusion coefficient <Deff> of 200 nm NPs was greatly increased in the PMB^Sens^ IR57 biofilms (0.0643 vs. 0.34 cm^2^ s^−1^ × 10^−9^) following treatment, while NP diffusion within the PMB^R^ PN47 biofilms remained largely unchanged (Table 2). This trend was reflected in the heterogeneity of NP diffusion measurements (Fig. 6), where the PMB^Sens^ IR57 biofilm demonstrated decreased 90th/10th percentile ratio of 2577 to 198 following treatment, revealing more homogenous NP diffusion, indicative of increasing biofilm pore size with antibiotic treatment. As before, the heterogeneity of the NP diffusion measurements within the PMB^R^ PN47 biofilms demonstrated little response to treatment. This result was also confirmed by the creep compliance data showing increasing values following polymyxin B treatment in the PMB^Sens^ strain, but not in the PMB^R^ strain (Fig. 7). Interestingly, NP diffusion (<Deff>) within the PMB^Sens^ IR57 biofilm was more than three times greater than the <Deff> for PMB^R^ PN47 biofilm (Table 2). NP diffusion within the PMB^Sens^ strain appeared to be vastly more heterogeneous than for the PMB^R^ strain, indicating that pore size was more heterogeneous in the biofilms of the PMB^Sens^ strain (Fig. 6).

**Table 2:** NP diffusion within PMB^R^ and PMB^sens^ *E. coli* biofilms ± polymyxin B treatment.

*E. coli* strain	Polymyxin B treatment (8 µg ml^−1^)	Diffusion coefficient in water (D°) (cm^2^/s × 10^−9^)	Diffusion coefficient <Deff>_*E. coli*_ (cm^2^/s × 10^−9^) Mean (±SEM)	% ratio <Deff>/D°
PMB^sens^ IR57	−	18.77	0.0643 (±0.0158)	0.3426
	+	18.77	0.3400 (±0.0805)	1.8114
PMB^R^ PN47	−	18.77	0.0198 (±0.0038)	0.1055
	+	18.77	0.0137 (±0.0025)	0.0730

Diffusion coefficients <Deff> of 200 nm negatively charged carboxylate-modified *Fluo*Spheres® in water (calculated by Stoke–Einstein equation) versus their effective diffusion coefficients (measured by MPT) through PMB^R^ and PMB^sens^ *E*. co*li* biofilms with***/***without polymyxin B treatment (8 µg/ml).

<Deff>_*E. coli*_ indicates <Deff> in PMB^R^ and PMB^sens^ *E. coli* biofilms.

± represents standard error of the mean (SEM; *n* = 3).

## Discussion

NPs are being increasingly applied in a variety of medical applications, such as in the diagnosis and treatment of human disease (e.g. drug- and gene delivery)^41^ and as novel research tools (both in vitro and in vivo) to improve our understanding of biological systems and human illness^42–44^. This study has demonstrated the use of MPT with traceable NPs as a robust, non-invasive, in situ technique to inform our understanding of biofilms and further our insight into the potential effects of antimicrobial and anti-biofilm therapies on the biofilm matrix, and on biofilm-related changes induced with the acquisition of antibiotic resistance.

The EPS matrix of biofilms varies not only between species and strains, but also with differences in environmental growth conditions, such as surface composition/roughness, nutrient availability, temperature, and hydrodynamic shear^38,45^. As a result, the net charge and functional groups present within the matrix, as well as biofilm pore size, exhibit considerable variations. To assess variations in biofilm structure between bacterial species, the diffusion coefficients of fluorescently-labelled NPs were initially measured in both Gram-positive *S. aureus* 1004A (MRSA) and Gram-negative *P. aeruginosa* PAO1, revealing the influence of both NP size and surface-charge on diffusion through the respective biofilm structures. The influence of NP charge was clearly evident in the diffusion coefficient measurements of 200 nm NPs within *P. aeruginosa* PAO1 biofilms, with reduced diffusion of amine-modified (positively charged) particles compared to the carboxylate-modified (negatively charged) NPs. In *P. aeruginosa* PAO1, the exopolysaccharides form a major part of the EPS matrix and are composed of negatively charged alginate, neutrally-charged Psl and positively charged Pel, while eDNA in biofilm matrixes has been shown to be negatively charged^46^. Our results indicate a net negative charge of the *P. aeruginosa* EPS matrix, where the existence of increased negative charges within the EPS matrix reduced the diffusion coefficient of the positively charged amine-modified NPs. In *S. aureus* MRSA biofilms, while the diffusion coefficient of the amine-modified NPs was twice that of the carboxylated-modified NPs, these values were not significantly different. In *S. aureus*, the two major components of EPS matrix are the positively charged poly-N-acetylgluosamine (PNAG) polysaccharides and negatively charged eDNA^47,48^. These results potentially indicate a net positive charge of the MRSA EPS matrix; increased positive charges within the matrix reducing the diffusion coefficient of the negatively charged carboxylated NPs. However, as the diffusion coefficient values achieved with 200 nm carboxylate-modified NPs were similar to those observed with 500 nm NPs in MRSA (0.0031 vs 0.0038 cm^2^ S^−1^ × 10^−9^, respectively), this indicated the inability of the 200 nm NPs to freely move within the dense MRSA biofilm matrix. The restricted movement of 200 nm NPs within the MRSA biofilms resulted in small, distinct differences in diffusion coefficient between the amine-modified NPs and carboxylate-modified NPs, which may have been more pronounced with the use of smaller sized amine-modified NPs.

In this model, NP diffusion into the biofilm EPS matrix was not only clearly influenced by charge, but also by their size^49–51^. Large particles may not be able to permeate into the biofilm EPS structure due to steric hindrance or biofilm pore size, while hydrophobic/electrostatic interactions may influence the diffusion of smaller particles^38^. Previous researchers have used a variety of techniques to characterise the internal structure and pore size of biofilms. Zhang et al.^52^ originally employed sectioning by microtome and dye adsorption to describe the heterogeneity in pore size within wastewater biofilms, where biofilm pore sizes ranged between 0.3 and 2.7 µm. More recently, Rosenthal et al.^53^ used optical coherence tomography (OCT) to characterize the heterogeneous network of a thick, multi-species biofilm, where pore diameters as large as 110 µm were measured, while other researchers have demonstrated pore-size ranges of 500–1000 nm^54^ and >100 nm^55^ using single-particle tracking techniques.

In the current study, the effect of NP size on diffusion was clearly evident in PAO1 and MRSA biofilms, where a reduction in NP size (<200 nm), induced a dramatic rise in particle diffusion; a finding possibly indicative of a mean biofilm pore size <200 nm. Similarly, Peulen and Wilkinson^56^ demonstrated increasing diffusion coefficients with decreasing size and negative charge of silver NPs in *Pseudomonas fluorescens* biofilms, where the optimum particle size for diffusion in these dense biofilms was <50 nm. Increased NP diffusion coefficients in PAO1 biofilms when compared to the MRSA biofilms (40–500 nm carboxylate-modified NPs) were evident in this study and reflected the biofilm architecture; CLSM imaging demonstrating the more dense structure of the MRSA biofilms.

Profiling of the diffusion coefficients of 360 individual particles through ranking <Deff> data into percentiles (highest 90th to lowest 10th percentiles) provides insight into the heterogeneity of particle movement, which may be indicative of heterogeneous pore sizes within the biofilm itself^57,58^. This study revealed that NP diffusion coefficients became more heterogeneous with increasing particle size until a NP size of 100 nm for *S. aureus* and 200 nm for *P. aeruginosa* biofilms was reached, at which point NP diffusion became more homogeneous (the particle size becoming too large to facilitate free movement within the biofilm structure) with NP movement occurring only through larger pore sizes. This data demonstrates contrasting inter-species biofilm architecture; *P. aeruginosa* forming a pore structure that was larger in size and more heterogeneous compared to that of *S. aureus* biofilms.

MPT may, in the future, offer valuable insight in characterizing the EPS matrix of heterogeneous polymicrobial biofilms which are commonly found in human disease states^59,60^. While these biofilms are heterogeneous, they are often composed of distinct, individual ‘pockets’ of homogenous single-species growth and MPT coupled with GFP-labelled bacterial populations or fluorescence in situ hybridization (FISH) labelling could be employed to visualize and analyse the biofilm matrix of these bacterial populations.

Structurally, biofilms possess a complex architecture, being composed of cell clusters surrounded by voids and water channels^61^. Biofilm structures possess viscoelastic properties (exhibiting both elastic and viscous properties) which can aid biofilm survival under mechanical and chemical loads^62,63^. Cao et al.^37^ employed single-particle tracking with CLSM imaging to characterize the viscoelastic properties of cell clusters and voids within *P. fluorescens* biofilms, demonstrating that the viscoelastic properties (creep compliance) of the ‘biofilm void’ zones was the primary contributor to the viscoelastic properties of the biofilm. Importantly, while the larger (500 nm) NPs employed in this study were unable to diffuse into the bacterial cell clusters, their diffusion into the biofilm voids could still provide a sensitive and reproducible model to monitor changes in the viscoelastic properties of biofilms. In this study, calculation of the exponential anomalous values revealed that PAO1 and MRSA biofilms displayed viscous behaviour when assessed using 40–500 nm NPs, however, no micro-rheological differences could be determined between the well-established 72 h grown PAO1 and MRSA biofilms.

The resistance of bacterial biofilms to antibiotic therapy has been widely reported in the literature, with high levels of antibiotic tolerance arising due to a number of different factors^64^. Penetration of antibiotics through the biofilm EPS matrix can vary considerably depending on how they interact with the charged components of the EPS^10,11^. Previous research has shown that cationic antibiotics (e.g. tobramycin) exhibit charge-mediated binding to polyanions within the biofilm EPS matrix, resulting in reduced penetration of antibiotics into the biofilm structure^9,65^. Sankaran et al.^34^, however, demonstrated that both labelled aminoglycoside tobramycin (positively-charged) and the fluoroquinolone ciprofloxacin (neutrally-charged) were able to diffuse into, and remained mobile within the interior of *P. aeruginosa* biofilms, demonstrating that reduced antibiotic diffusion in the EPS matrix is not solely responsible for the antibiotic tolerance of biofilms^66^. Moreover, the development of a sub-population of persister cells (with low or dormant metabolic activity) may ensure that a small cohort of cells within the biofilm can withstand multiple doses of antimicrobial therapy^67^. Expression of biofilm-specific genetic mechanisms may also occur^11^, making the biofilm less susceptible to antimicrobial attack.

Zrelli et al.^68^ and Reighard et al.^39^ demonstrated the failure of antibiotic treatment (afloxacin, ticarcillin, tobramycin) to induce changes in the NP diffusion and mechanical properties of *E. coli* and *P. aeruginosa* biofilms using particle tracking methodologies. In contrast, here we demonstrate the dose-dependent disruption of the PMB^sens^ *E. coli* biofilm matrix induced by exposure to polymyxin B, with significant increases in NP diffusion and creep compliance in the treated biofilms. Klinger-Strobel et al.^69^ demonstrated the ability of colistin (polymyxin E) to disrupt 48 h *E. coli* biofilms at concentrations of 4–16 µg/ml (with MICs ranging from 1 to 0.0625 µg/ml for laboratory strains and clinical isolates, respectively). The authors suggested that colistin destabilized the biofilm matrix (even in strains with intrinsic polymyxin resistance) leading to the dispersal of planktonic cells that were then more susceptible to antibiotics. The published susceptibility breakpoints for polymyxins are S ≤ 2 µg/ml > R for *E. coli* (EuCAST 2018)^70^. The concentrations of polymyxin B used in our study were relatively high (2–64 µg/ml), equal to, or above, the MIC values (0.06–2 µg/ml), to induce effective disruption of the biofilm structure. However, differences in NP diffusion were detected at lower concentrations (2 µg/ml) than that employed by Klinger-Strobel et al.^69^.

Witten and Ribbeck^38^ proposed that biofilm permeability may be a biomarker for antimicrobial resistance, and the data here demonstrates the sensitivity of the MPT biofilm assay in this respect. In the PMB^sens^ *E. coli* IR57 (MIC 0.06 µg/ml) biofilms, MPT was able to detect structural, pore-size related changes induced within the biofilm matrix with antibiotic dosing as low as 2 µg/ml (a clinically-relevant concentration) where changes in the treated biofilm structure were indiscernible at this concentration using conventional CLSM imaging. The disruptive effect of polymyxin B on the *E. coli* biofilm structure was, however, evident in CLSM imaging at higher concentrations (>32 µg/ml). We also sought to model biofilm disruption by polymyxin B in PMB^R^ *E. coli* as we have previously demonstrated that *mcr-1* and *-3* expression in *E. coli* is associated with alterations in bacterial viability within the biofilm matrix^16,71^, hypothesizing that the acquisition of polymyxin resistant *mcr-1* was an evolutionary ‘trade-off’; resistance to polymyxin being associated with decreased biofilm biomass and growth in 24 h biofilm formation assays^71^. Here, we clearly demonstrate the resistance to biofilm disruption of PMB^R^ PN47 following treatment with polymyxin B (8 µg/ml) in well-established 72 h grown biofilms.

The observed variations in particle diffusion and creep compliance with antibiotic/antimicrobial treatment in this study could also be crucial in understanding the interaction of the innate immune system with biofilm infections. Increased pore size and decreased mechanical properties of the biofilm structure may facilitate increased inflammatory cell penetration into the biofilm matrix, thereby improving bacterial clearance^72^. Moreover, increased porosity of biofilm structures may allow greater penetration of antimicrobial agents and may be of clinical benefit, especially in treating implant biofilm-related infections^73^.

This study demonstrated not only the ability of MPT to detect disruption in biofilm structures, but also revealed the ability of the technique to dissect the effects of antimicrobial and anti-biofilm therapies, not always discernible using conventional technologies. Here, the ability of MPT to inform understanding of, and test therapies against, emergent antimicrobial-resistant pathogens is clear. As anti-biofilm therapies play a clear role in increasing susceptibility of resistant bacteria to existing therapies, the utility of MPT in assessing novel therapies to disrupt the biofilm matrix, e.g. chelating agents^23^ and G-block alginate oligomers^31,74^ may be invaluable in the future. MPT may further our insight into the potential effects of such antimicrobial therapies in vivo and provide increased understanding into the biofilm matrix and biofilm-related changes induced with the acquisition of antibiotic resistance.

## Methods

### Bacterial strains, growth media and culture conditions

The following strains were used in this study; *Pseudomonas aeruginosa* PAO1, methicillin-resistant *Staphylococcus aureus* (MRSA 1004A)^75^, polymyxin B-resistant (PMB^R^) *Escherichia coli* PN47 (carrying the colistin resistance plasmids *mcr-1* and *mcr-3*)^76^ and polymyxin B-sensitive (PMB^Sens^) *E. coli* IR57. Bacterial colonies were sub-cultured on LB agar plates supplemented with/without 2 µg/ml polymyxin B (Sigma-Aldrich). Overnight bacterial cultures were grown in Tryptone Soy Broth (TSB; Oxoid) for 37 °C at 120 rpm. Biofilms were grown in cation-adjusted Mueller Hinton Broth (MHB; LabM) with/without supplementation with the antibiotic polymyxin B.

### Minimum inhibitory concentration (MIC) measurements

Overnight bacterial cultures were adjusted to a standardized cell suspension of ~10^8^ colony forming units (CFU)/ml (equivalent to 0.5 McFarland standard). Two-fold serial dilutions in polymyxin B were prepared in MHB within flat-bottom 96-well microtiter plates (100 µl per well). The adjusted O/N bacterial cultures were then diluted 10-fold in MHB and 5 µl added to the microtiter plate containing the antibiotic serial dilutions to give a final concentration of 5 × 10^5^ CFU/ml. The plates were incubated for 16–20 h (37 °C) and MICs determined as the lowest concentration at which there was no visible growth.

### Biofilm growth

Overnight bacterial cultures were adjusted to a standardized cell suspension of 1 × 10^7^ CFU/ml in MHB. First, 0.2 ml of adjusted O/N culture was placed into the centre of each glass well within a 12-well dish (glass thickness 1.5 and 14 mm diameter; MatTek) and incubated statically for 1 h. Then, 1.8 ml of fresh MHB was placed into each well and incubated statically for 24 h at 37 °C. A further 1 ml of MHB was then placed into each well and plates incubated for a further 24 h (37 °C). Following 48 h growth, biofilms were treated with a further 1 ml of MHB which was added into each well with a further incubation of 24 h (37 °C), resulting in a total biofilm growth time of 72 h.

### Polymyxin B treatment of *E. coli* biofilms

Following 48 h growth, *E. coli* biofilms were treated with either 1 ml of MHB (control) or 1 ml polymyxin B (2, 8, 16, 32, 64 µg/ml; treatment) which was added into each well with a further incubation of 24 h (37 °C), resulting in a total biofilm growth time of 72 h.

### SYTO 9 staining and CLSM imaging of biofilms

After 72 h growth, the bacterial supernatant was carefully removed and the bacterial cells within the biofilms stained with 0.5% Syto9® dye (Invitrogen; 400 µl) for 1 h. After staining, the biofilms were washed with phosphate-buffered saline (PBS; x2) prior either to CLSM Z-stack imaging using a Leica TCS SP5 CLSM or NP addition.

### MPT measurement of bacterial biofilms

NPs used in this study were either negatively charged carboxylate-modified *Fluo*spheres® (40, 100, 200 and 500 nm) or positively charged amino-modified *Fluo*spheres® (200 nm) ([Ex/Em]: [580/605 nm]; ThermoFisher Scientific). The independent assessment of size and zeta potential values of the NPs were characterized in PBS buffer using a Malvern Zetasizer Nano ZS prior to MPT studies. For MPT experiments, the *Fluo*spheres® suspension was vortexed for 1 min then diluted in sterilized PBS buffer (0.0025%), before addition of 10 µl diluted *Fluo*spheres® suspension onto the biofilms followed by a 2 h incubation. Biofilms were stained with SYTO 9® before addition of the *Fluo*spheres® to visualize the lower layers of the biofilm matrix using a Leica DM IRB wide-field Epifluorescence microscope (×63 oil immersion lens). Videos of particle movement within the biofilms were captured at a frame rate of 33 ms (600 frames, 20 s) using a high-speed camera (Allied Vision Technologies, UK) and then particle trajectories were tracked using ImageJ software (Mosaic) over 2 s to convert NP movements into metric displacements in both the X and Y directions^43,77^. Ensemble mean square displacement <MSD>, effective diffusion coefficient <Deff>, and heterogeneity of particle diffusion were measured as described in supplementary materials^57,78^. Here, 120 particle movements were captured in each biofilm well, and each bacterial strain was tested in triplicate (i.e. 360 particles in total for each biofilm species).

The viscoelastic properties of the biofilms were assessed by determining the anomalous diffusion exponent (*α*). This was calculated by fitting the power law to log(<MSD>) versus log(Δ*t*) and calculating the slope of this data^79^, where *α* = 1 for a completely viscous system (liquid), *α* = 0 for a completely elastic system (solid) and 1 > *α* > 0 for a viscoelastic system^39,40^. In addition, the micro-rheological properties of the biofilms were further defined by calculation of the creep compliance (*J*(*t*)), where the MSD represents deformation of the biofilm within time (Δ*t*) under constant pressure/shear force represented by the temperature of the atmosphere. Creep compliance (*J*(*t*)) was calculated by the following equation:

$$J(t) = \frac{{3\pi d}}{{4k_{\mathrm{B}}T}}{\mathrm{MSD}}(t)$$

where *k*_B_ is the Boltzmann constant, *T* is absolute temperature and *d* is the diameter of the particle^80^.

### Statistical analysis

Statistical software (Minitab, State College, PA) was used to calculate significant differences with ANOVA testing with post hoc Tukey multi-comparison tests for the statistical analyses presented.

### Reporting summary

Further information on research design is available in the Nature Research Reporting Summary linked to this article.

## Supplementary information

Supplementary Information

Reporting Summary

## Data Availability

Data generated and analysed during this study are included in this published article and its Supplemental information file. Additional details are available upon reasonable request.

## References

[1] Bjarnsholt T (2013). The role of bacterial biofilms in chronic infections. APMIS Suppl..

[2] Mazza MG (2016). The physics of biofilms—an introduction. J. Phys. D. Appl. Phys..

[3] Flemming H-C (2016). Biofilms: an emergent form of bacterial life. Nat. Rev. Microbiol..

[4] Boudarel H, Mathias J-D, Blaysat B, Grediac M (2018). Towards standardized mechanical characterization of microbial biofilms: analysis and critical review. NPJ Biofilms Microbiomes.

[5] Mah TF (2012). Biofilm-specific antibiotic resistance. Future Microbiol..

[6] Ceri H (1999). The Calgary biofilm device: new technology for rapid determination of antibiotic susceptibilities of bacterial biofilms. J. Clin. Microbiol..

[7] Moskowitz SM, Foster JM, Emerson J, Burns JL (2004). Clinically feasible biofilm susceptibility assay for isolates of *Pseudomonas aeruginosa* from patients with cystic fibrosis. J. Clin. Microbiol..

[8] Høiby N, Bjarnsholt T, Givskov M, Molin S, Ciofu O (2010). Antibiotic resistance of bacterial biofilms. Int. J. Antimicrob. Agents.

[9] Tseng BS (2013). The extracellular matrix protects *Pseudomonas aeruginos*a biofilms by limiting the penetration of tobramycin. Environ. Microbiol..

[10] Hunt BE, Weber A, Berger A, Ramsey B, Smith AL (1995). Macromolecular mechanisms of sputum inhibition of tobramycin activity. Antimicrob. Agents Chemother..

[11] Mah TF (2003). A genetic basis for *Pseudomonas aeruginosa* biofilm antibiotic resistance. Nature.

[12] Lindsay JA (2013). Hospital-associated MRSA and antibiotic resistance—what have we learned from genomics?. Int J. Med. Microbiol..

[13] Corona A, Cattaneo D (2017). Dosing colistin properly: let’s save ‘Our Last Resort Old Drug. Clin. Infect. Dis..

[14] Bulman ZP (2017). Polymyxin combinations combat *Escherichia coli* harboring *mcr-1* and blaNDM-5: preparation for a post-antibiotic era. MBio.

[15] Poirel L (2018). Antimicrobial resistance in *Escherichia coli*. Microbiol. Spectr..

[16] Yang Q (2017). Balancing *mcr-1* expression and bacterial survival is a delicate equilibrium between essential cellular defense mechanisms. Nat. Commun..

[17] Zhao F, Feng Y, Lü X, Mcnally A, Zong Z (2017). IncP plasmid carrying colistin resistance gene *mcr-1* in *Klebsiella pneumoniae* from hospital sewage. Antimicrob. Agents Chemother..

[18] Lemire JA, Harrison JJ, Turner RJ (2013). Antimicrobial activity of metals: mechanisms, molecular targets and applications. Nat. Rev. Micro.

[19] Bayramov DF, Neff JA (2016). Beyond conventional antibiotics—new directions for combination products to combat biofilm. Adv. Drug Deliv. Rev..

[20] Pletzer D, Coleman SR, Hancock RE (2016). Anti-biofilm peptides as a new weapon in antimicrobial warfare. Curr. Opin. Microbiol..

[21] Barraud N, Kelso MJ, Rice SA, Kjelleberg S (2015). Nitric oxide: a key mediator of biofilm dispersal with applications in infectious diseases. Curr. Pharm. Des..

[22] Otzen DE (2017). Biosurfactants and surfactants interacting with membranes and proteins: same but different. BBA: Biomembranes.

[23] Finnegan S, Percival SL (2015). EDTA: an antimicrobial and antibiofilm agent for use in wound care. Adv. Wound Care.

[24] Pandit S (2017). Low concentrations of Vitamin C reduce the synthesis of extracellular polymers and destabilize bacterial biofilms. Front. Microbiol..

[25] Jack AA (2018). Alginate oligosaccharide-induced modification of the lasI-lasR and rhlI-rhlR quorum sensing systems in *Pseudomonas aeruginosa*. Antimicrob. Agents Chemother..

[26] Brackman G, Coenye T (2015). Quorum sensing inhibitors as anti-biofilm agents. Curr. Pharm. Des..

[27] Pan M, Zhu L, Chen L, Qiu Y, Wang J (2016). Detection techniques for extracellular polymeric substances in biofilms: a review. BioRes.

[28] Jennings LK (2015). Pel is a cationic exopolysaccharide that cross-links extracellular DNA in the *Pseudomonas aeruginosa* biofilm matrix. Proc. Natl Acad. Sci. USA..

[29] Billings N (2013). The extracellular matrix component Psl provides fast-acting antibiotic defense in *Pseudomonas aeruginosa* biofilms. PLoS Pathog..

[30] Franklin MJ, Nivens DE, Weadge JT, Howell PL (2011). Biosynthesis of the *Pseudomonas aeruginosa* extracellular polysaccharides, alginate, Pel, and Psl. Front. Microbiol..

[31] Powell LC (2013). The effect of alginate oligosaccharides on the mechanical properties of Gram-negative biofilms. Biofouling.

[32] Pritchard MF (2017). A low-molecular-weight alginate oligosaccharide disrupts pseudomonal microcolony formation and enhances antibiotic effectiveness. Antimicrob. Agents Chemother..

[33] Gulot E (2002). Heterogeneity of diffusion inside microbial biofilms determined by fluorescence correlation spectroscopy under two-photon excitation. Photochem Photobio..

[34] Sankaran J (2019). Single microcolony diffusion analysis in *Pseudomonas aeruginosa* biofilms. NPJ Biofilms Microbiomes.

[35] Waharte F, Steenkeste K, Briandet R, Fontaine-Aupart MP (2010). Diffusion measurements inside biofilms by image-based fluorescence recovery after photobleaching (FRAP) analysis with a commercial confocal laser scanning microscope. Appl. Environ. Microbiol..

[36] Billings N, Birjiniuk A, Samad TS, Doyle PS, Ribbeck K (2015). Materials properties of biofilms—a review of methods for understanding permeability and mechanics. Rep. Prog. Phys..

[37] Cao H (2016). Revealing region-specific biofilm viscoelastic properties by means of a micro-rheological approach. NPJ Biofilms Microbiomes.

[38] Witten J, Ribbeck K (2017). The particle in the spider’s web: transport through biological hydrogels. Nanoscale.

[39] Reighard KP, Hill DB, Dixon GA, Worley BV, Schoenfisch MH (2015). Disruption and eradication of P. aeruginosa biofilms using nitric oxide-releasing chitosan oligosaccharides. Biofouling.

[40] Chew SC, Rice SA, Kjelleberg S, Yang L (2015). In situ mapping of the mechanical properties of biofilms by particle-tracking microrheology. J. Vis. Exp..

[41] Zazo H, Colino CI, Lanao JM (2016). Current applications of nanoparticles in infectious diseases. J. Control Release.

[42] Natan M, Banin E (2017). From nano to micro: using nanotechnology to combat microorganisms and their multidrug resistance. FEMS Microbiol. Rev..

[43] Inchaurraga L (2019). Modulation of the fate of zein nanoparticles by their coating with a Gantrez® AN-thiamine polymer conjugate. Int J. Pharm. X.

[44] Brotons-Canto A (2018). Evaluation of nanoparticles as oral vehicles for immunotherapy against experimental peanut allergy. Int. J. Biol. Macromol..

[45] Fulaz S, Vitale S, Quinn L, Casy E (2019). Nanoparticle-biofilm interactions: the role of the EPS matrix. Trends Microbiol..

[46] Jennings LK (2015). Pel is a cationic exopolysaccharide that cross-links extracellular DNA in the *Pseudomonas aeruginosa* biofilm matrix. PNAS.

[47] Dengler V, Foulston L, DeFrancesco A, Losick R (2015). An electrostatic net model for the role of extracellular DNA in biofilm formation by *Staphylococcus aureus*. J. Bacteriol..

[48] Hiltunen AK (2019). Structural and functional dynamics of *Staphylococcus aureus* biofilms and biofilm matrix proteins on different clinical materials. Microorganisms.

[49] Campoccia D, Montanaro L, Arciola CR (2013). A review of the biomaterials technologies for infection-resistant surfaces. Biomaterials.

[50] Habimana O, Steenkeste K, Fontaine-Aupart MP, Bellon-Fontaine M-N, Briandet R (2011). Diffusion of nanoparticles in biofilms is altered by bacterial cell wall hydrophobicity. Appl. Environ. Microbiol..

[51] Ikuma K, Decho AW, Lau BLT (2015). When nanoparticles meet biofilms—interactions guiding the environmental fate and accumulation of nanoparticles. Front. Microbiol..

[52] Zhang TC, Bishop PL (1994). Density, porosity and pore structure of biofilms. Water Res..

[53] Rosenthal AF (2018). Morphological analysis of pore size and connectivity in a thick mixed culture biofilm. Biotechnol. Bioeng..

[54] Chew SC (2014). Dynamic remodeling of microbial biofilms by functionally distinct exopolysaccharides. MBio.

[55] Forier K (2013). Transport of nanoparticles in cystic fibrosis sputum and bacterial biofilms by single-particle microscopy. Nanomedicine.

[56] Peulen TO, Wilkinson KJ (2011). Diffusion of nanoparticles in a biofilm. Environ. Sci. Technol..

[57] Abdulkarim M (2015). Nanoparticle diffusion within intestinal mucus: three-dimensional response analysis dissecting the impact of particle surface charge, size and heterogeneity across polyelectrolyte, pegylated and viral particles. Eur. J. Pharm. Biopharm..

[58] Sahle-Demessie E, Tadesse H (2011). Kinetics and equilibrium adsorption of nano-TiO_2_ particles on synthetic biofilm. Surf. Sci..

[59] Zijnge V (2010). Oral biofilm architecture on natural teeth. PLoS ONE.

[60] Bjarnsholt T (2008). Why chronic wounds will not heal: a novel hypothesis. Wound Repair Regen..

[61] Donlan RM, Costerton JW (2002). Biofilms: survival mechanisms of clinically relevant microorganisms. Clin. Microb. Rev..

[62] Peterson BW (2015). Viscoelasticity of biofilms and their recalcitrance to mechanical and chemical challenges. FEMS Microbiol. Rev..

[63] Rogers SS, van der Walle C, Waigh TA (2008). Microrheology of bacterial biofilms in vitro: *Staphylococcus aureus* and *Pseudomonas aeruginosa*. Langmuir.

[64] Lebeaux D, Ghigo J-M, Beloin C (2014). Biofilm-related infections: bridging the gap between clinical management and fundamental aspects of recalcitrance toward antibiotics. Microbiol. Mol. Biol. Rev..

[65] Huang JX (2015). Mucin binding reduces colistin antimicrobial activity. Antimicrob. Agents Chemother..

[66] Walters MC, Roe F, Bugnicourt A, Franklin MJ, Stewart PS (2003). Contributions of antibiotic penetration, oxygen limitation, and low metabolic activity to tolerance of *Pseudomonas aeruginosa* biofilms to ciprofloxacin and tobramycin. Antimicrob. Agents Chemother..

[67] Percival SL, Hill KE, Malic S, Thomas DW, Williams DW (2011). Antimicrobial tolerance and the significance of persister cells in recalcitrant chronic wound biofilms. Wound Repair Regen..

[68] Zrelli K (2013). Bacterial biofilm mechanical properties persist upon antibiotic treatment and survive cell death. N. J. Phys..

[69] Klinger-Strobel M, Stein C, Forstner C, Makarewicz O, Pletz MW (2017). Effects of colistin on biofilm matrices of *Escherichia coli* and *Staphylococcus aureus*. Int J. Antimicrob. Agents.

[70] EUCAST. Breakpoint tables for interpretation of MICs and zone diameters. http://www.eucast.org/fileadmin/src/media/PDFs/EUCAST_files/Breakpoint_tables/v_8.1_Breakpoint_Tables.pdf (2018).

[71] Yang QE (2020). Compensatory mutations modulate the competitiveness and dynamics of plasmid-mediated resistance in *Escherichia coli* clones. ISME J..

[72] Geddes-McAlister, J., Kugadas, A. & Gadjeva, M. Tasked with a challenging objective: Why do neutrophils fail to battle *Pseudomonas aeruginosa* biofilms. *Pathogens* **8**, 283 (2019). 10.3390/pathogens8040283 PMC6963930 31817091

[73] Connaughton A, Childs A, Dylewski S, Sabesan VJ (2014). Biofilm disrupting technology for orthopedic implants: what’s on the horizon?. Front. Med..

[74] Powell LC (2018). Targeted disruption of the extracellular polymeric network of *Pseudomonas aeruginosa* biofilms by alginate oligosaccharides. NPJ Biofilms Microbiomes.

[75] Howell-Jones, R. S. *Antibiotic use in the Treatment of Chronic Wounds*. PhD Thesis, Cardiff University (2007).

[76] Yang QE (2018). Environmental dissemination of *mcr-1* positive Enterobacteriaceae by *Chrysomya* spp. (common blowfly): an increasing public health risk. Environ. Int.

[77] Bonengel S (2018). Impact of different hydrophobic ion pairs of octreotide on its oral bioavailability in pigs. J. Control Release.

[78] Rohrer J (2016). Mucus permeating thiolated self-emulsifying drug delivery systems. Eur. J. Pharm. Biopharm..

[79] Schuster BS, Suk JS, Woodworth GF, Hanes J (2013). Nanoparticle diffusion in respiratory mucus from humans without lung disease. Biomaterials.

[80] Birjiniuk A (2014). Single particle tracking reveals spatial and dynamic organization of the *Escherichia coli* biofilm matrix. N. J. Phys..

