# Supplementary material for: Quantifying the effects of antibiotic treatment on the extracellular polymer network of antimicrobial resistant and sensitive biofilms using multiple particle tracking
Source: NPJ Biofilms Microbiomes. 2021 Feb 5;7:13. doi: 10.1038/s41522-020-00172-6 (PMC7864955; doi:10.1038/s41522-020-00172-6)
Supplement: Supplementary file 1 — Supplementary Information [file 41522_2020_172_MOESM1_ESM.pdf]

SUPPLEMENTARY MATERIALS:

Quantifying the effects of antibiotic treatment on the extracellular polymer network of antimicrobial resistant and sensitive biofilms using multiple particle tracking.

Lydia C. Powell, Muthanna Abdulkarim, Joana Stokniene, Qiu E. Yang, Timothy R. Walsh, Katja E. Hill, Mark Gumbleton, David W. Thomas

SUPPLEMENTARY FIGURE

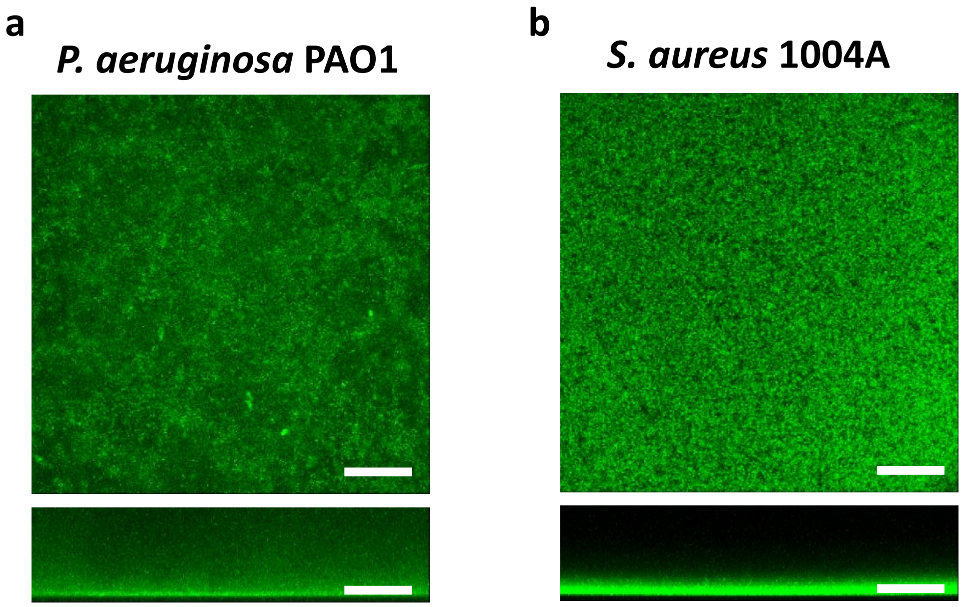

Supplementary Fig. 1. Comparison *of P. aeruginosa* and *S. aureus* biofilm structures. CLSM 3D imaging of (a) *P. aeruginosa* PAO1 and (b) *S. aureus* 1004A (MRSA) biofilms grown for 72 h at 37°C in MH broth, using Syto9® staining (Scale bar, 40 µm; n=3).

SUPPLEMENTARY MATERIALS AND METHODS

P. aeruginosa bacterial strain.

P. aeruginosa PAO1 used in this study closely resembles PAO1_Orsay and PAO1_ ATCC 15692 strains, as demonstrated in high-throughput genome resequencing in an unrelated study (European Nucleotide Archive[ENA] project number PRJEB36146 and accession number for the draft genome sequence GCA_902860215).

Calculation of mean square displacement ‹MSD›, effective diffusion coefficient ‹Deff›, and heterogeneity of particle diffusion

The mean square displacement ‹MSD› was determined as follows:

$$\mathrm{MSD}_{(n)} = (\mathrm{X}_{\Delta t})^2 + (\mathrm{Y}_{\Delta t})^2 \quad (1)$$

where the distance the nanoparticle (n) moved over a selected time frame (t) in the X-Y trajectory was expressed as a squared displacement (SD).

The effective diffusion coefficient ‹Deff›, of the nanoparticles determined by the following equation:

$$\langle \mathrm{Deff} \rangle = \langle \mathrm{MSD} \rangle / (4 * \Delta t) \quad (2)$$

where 4 is a constant relating to the 2-dimensional mode of video capture and Δt is the selected time interval.

Nanoparticle diffusion in water (D°) was calculated by the Stokes–Einstein equation at 37 ^{o}C:

$$\mathrm{D}^{\circ} = k_B \mathrm{T}/6\pi\eta r \quad (3)$$

where k_B is the Boltzmann constant, T is absolute temperature, η is water viscosity, and r is

radius of the nanoparticle.

The diffusion of the nanoparticles was also expressed as the parameter, % ratio [Deff]/[D°]. The heterogeneity of particle diffusion was measured by profiling the diffusion coefficients (Δt= 2 sec) of all individual particles within the entire population (360 particles) from the highest (90th) to the lowest (10th) percentiles in ‹Deff› values.

Error arising from experimental noise

The error arising from experimental noise (tracking resolution [σ]) was measured for each of the *Fluo*Sphere particles individually, by fixing the particles onto a glass-bottomed imaging dish (MatTek life sciences) with cyanoacrylate-based glue and tracking their movements. Using this set-up, 20 videos were analysed using ImageJ software with Mosaic plugin to independently measure σ^2 by determining the X- and Y-directional displacement of the particles at the lowest temporal resolution (0.033 frame per second). The value of σ^2 was subtracted from the MSD measurement at the lowest frame rate to achieve final measurements of MSD and <Deff>. The calculated values of σ ranged between 3.45 to 3.96 nm for each of the *Fluo*spheres (Supplementary Table 1).

Supplementary Table 1. Tracking resolution (σ) of the *Fluo*Sphere® particles

***Fluo*Spheres®**	**Particle size (nm)**	**σ (nm)**
-ve carboxylate	40	3.96
-ve carboxylate	100	3.82
-ve carboxylate	200	3.45
-ve carboxylate	500	3.56
+ve amine	200	3.69
